# A 46-year-old man with recurrent embolic events

**DOI:** 10.1007/s12471-017-1034-8

**Published:** 2017-09-01

**Authors:** T. Lorjé, N. Barlo, C. L. A. Reichert, W. de Kanter, M. A. Sluman

**Affiliations:** 1 Department of Cardiology, Northwest Clinics, Alkmaar, The Netherlands; 2 Department of Pulmonology, Northwest Clinics, Alkmaar, The Netherlands; 3 Department of Pulmonology, Antoni van Leeuwenhoek Hospital, Amsterdam, The Netherlands; 4 Department of Internal Medicine, Alrijne Hospital, Leiderdorp, The Netherlands; 5 Department of Cardiology, Jeroen Bosch Hospital, ’s Hertogenbosch, The Netherlands

**Fig. 1:**
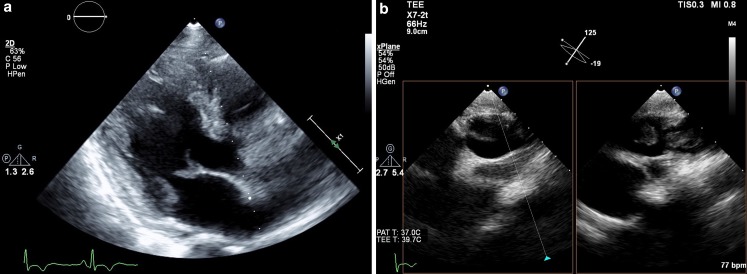
Transthoracic (**a**) and transoesophageal echocardiography (**b**) revealing a mobile structure attached to the ventricular side of the anterior mitral valve leaflet and multiple mobile masses at the origin of the pulmonary artery

A 46-year-old male with advanced adenocarcinoma of the lung presented with venous thrombosis. Despite anticoagulants, he developed a stroke and bowel ischaemia. Echocardiography revealed mobile structures on the mitral valve and at the pulmonary artery origin (Fig. 1). Although treatment for endocarditis and pulmonary embolism was initiated, his condition deteriorated. However, blood cultures remained negative and fever was absent. Non-bacterial thrombotic endocarditis (NBTE) became more likely after establishing progressive malignancy with PET-CT. NBTE is found in 19% of patients with malignancy and thrombotic events, but often not recognised [1]. It is distinguished from infective endocarditis by location of the valvular mass, absence of infection, diffuse thickening of valve leaflets and usual absence of valvular dysfunction [2, 3]. Treatment involves oncological therapy and anticoagulants [4]. Surgery may be considered in progressive valvular disease or recurrent embolism [5]. NBTE should be part of the differential diagnosis in patients with malignancy and thromboembolic complications.
